# Physical Interactions and Functional Relationships of Neuroligin 2 and Midbrain Serotonin Transporters

**DOI:** 10.3389/fnsyn.2015.00020

**Published:** 2016-01-11

**Authors:** Ran Ye, Meagan A. Quinlan, Hideki Iwamoto, Hsiao-Huei Wu, Noah H. Green, Christopher S. Jetter, Douglas G. McMahon, Jeremy Veestra-VanderWeele, Pat Levitt, Randy D. Blakely

**Affiliations:** ^1^Department of Pharmacology, Vanderbilt University School of Medicine, NashvilleTN, USA; ^2^Department of Psychiatry, Vanderbilt University School of Medicine, NashvilleTN, USA; ^3^Department of Biological Sciences, Vanderbilt University School of Medicine, NashvilleTN, USA; ^4^Department of Psychiatry, NYS Psychiatric Institute, Columbia University Medical Center, New YorkNY, USA; ^5^The Saban Research Institute, Children’s Hospital Los Angeles and University of Southern California, Los AngelesCA, USA

**Keywords:** serotonin, transporter, neuroligin2, neurexin, raphe nucleus

## Abstract

The neurotransmitter serotonin [5-hydroxytryptamine (5-HT)] modulates many key brain functions including those subserving sensation, emotion, reward, and cognition. Efficient clearance of 5-HT after release is achieved by the antidepressant-sensitive 5-HT transporter (SERT, *SLC6A4*). To identify novel SERT regulators, we pursued a proteomic analysis of mouse midbrain SERT complexes, evaluating findings in the context of prior studies that established a SERT-linked transcriptome. Remarkably, both efforts converged on a relationship of SERT with the synaptic adhesion protein neuroligin 2 (NLGN2), a post-synaptic partner for presynaptic neurexins, and a protein well-known to organize inhibitory GABAergic synapses. Western blots of midbrain reciprocal immunoprecipitations confirmed SERT/NLGN2 associations, and also extended to other NLGN2 associated proteins [e.g., α-neurexin (NRXN), gephyrin]. Midbrain SERT/NLGN2 interactions were found to be Ca^2+^-independent, supporting *cis* vs. *trans*-synaptic interactions, and were absent in hippocampal preparations, consistent with interactions arising in somatodendritic compartments. Dual color *in situ* hybridization confirmed co-expression of *Tph2* and *Nlgn2* mRNA in the dorsal raphe, with immunocytochemical studies confirming SERT:NLGN2 co-localization in raphe cell bodies but not axons. Consistent with correlative mRNA expression studies, loss of NLGN2 expression in *Nlgn2* null mice produced significant reductions in midbrain and hippocampal SERT expression and function. Additionally, dorsal raphe 5-HT neurons from *Nlgn2* null mice exhibit reduced excitability, a loss of GABA_A_ receptor-mediated IPSCs, and increased 5-HT_1A_ autoreceptor sensitivity. Finally, *Nlgn2* null mice display significant changes in behaviors known to be responsive to SERT and/or 5-HT receptor manipulations. We discuss our findings in relation to the possible coordination of intrinsic and extrinsic regulation afforded by somatodendritic SERT:NLGN2 complexes.

## Introduction

In the mammalian central nervous system (CNS), serotonin [5-hydroxytryptophan (5-HT)] acts as an essential modulatory neurotransmitter, regulating a wide variety of brain functions that range from sensory perception and pain responses to emotion and cognition, among many others (Azmitia, 2007). CNS serotonergic innervation originates from small clusters of mesencephalic and brainstem dorsal raphe neurons, with forebrain projections derived largely from dorsal and median raphe nuclei (Abrams et al., 2004; Gaspar and Lillesaar, 2012). In both somatodendritic and synaptic compartments, the availability of 5-HT is actively constrained by the high-affinity, antidepressant-sensitive 5-HT transporter (SERT, *SLC6A4*; Bunin and Wightman, 1998; Montanez et al., 2003; Mathews et al., 2004; Jennings et al., 2006). Alterations in SERT expression and function have been implicated in a range of neurobehavioral disorders including anxiety, depression, obsessive-compulsive disorder and autism (Lesch et al., 1996; Ozaki et al., 2003; Sutcliffe et al., 2005; Homberg and Lesch, 2011; Voyiaziakis et al., 2011). Although SERT proteins have been the target of therapeutic medications for decades, the endogenous mechanisms by which SERT levels and activity are adjusted to meet the ongoing demand for serotonergic neuromodulation remain to be fully elucidated (Steiner et al., 2008), with evidence that perturbed SERT regulation can contribute to disease risk (Ozaki et al., 2003; Sutcliffe et al., 2005; Baudry et al., 2010; Veenstra-VanderWeele et al., 2012).

SERT, like other neurotransmitter transporters (Sager and Torres, 2011), appears to be dynamically regulated by protein–protein interactions, though specific compartments and signals regulating the abundance and localization of these complexes are ill-defined, particularly *in vivo*. Recent studies from our group and others have revealed that SERT trafficking, activity and sensitivity to signaling pathways are influenced by an array of interacting proteins that include signaling enzymes (e.g., NOS1, PKG, PP2A; Bauman et al., 2000; Chanrion et al., 2007; Steiner et al., 2009), cell-surface receptors (e.g., A3AR and IL-1R; Zhu et al., 2010, 2011), and scaffolding and cell adhesion proteins (e.g., Hic-5, ITGB3; Carneiro and Blakely, 2006; Carneiro et al., 2008). Recently, in order to nominate novel regulators of SERT expression and 5-HT homeostasis, we implemented a gene-driven, bioinformatics approach based on mRNA correlations captured from midbrain transcriptomes of recombinant-inbred mice (BXD; Ye et al., 2014a,b). Attesting to the potential utility of this approach, we identified multiple genes whose expression is well-known to influence 5-HT neuron identity, synthesis and release, including the transcription factor gene *Fev* (also known as *Pet1*), the 5-HT biosynthetic enzyme tryptophan hydroxylase type 2 gene (*Tph2)*, and the vesicular monoamine transporter type 2 (VMAT2) gene (*Slc18a2)*. Additionally, we identified a number of genes whose relationships to SERT and 5-HT signaling have yet to be defined. One such gene (*Nlgn2*) encodes the *trans*-synaptic cell adhesion protein neuroligin 2 (NLGN2). We found that strain variation in *Nlgn2* mRNA levels exhibited a significant, positive correlation with strain variation of SERT (*Slc6a4*) and *Tph2* mRNA levels, suggesting that *Nlgn2* expression may be tightly coordinated with multiple dimensions of 5-HT homeostasis.

In an effort to obtain more direct insights into SERT regulatory machinery, we pursued a proteomic analysis of SERT-interacting proteins, defining specificity via parallel assessments of extracts from *Slc6a4* knockout mice. Remarkably, this effort yielded evidence of SERT:NLGN2 associations, as well as several other proteins known to associate with NLGN2 at inhibitory synapses. The convergence on NLGN2 of our prior SERT transcriptomic with this proteomic effort compelled a further evaluation of the relationship between transporter and synaptic adhesion protein. Our findings confirm specific SERT:NLGN2 protein associations. NLGN2 belongs to the neuroligin family, a family of integral membrane, synaptic adhesion proteins that possess an intracellular PDZ domain and an extracellular cholinesterase domain (Arac et al., 2007; Koehnke et al., 2008). NLGN2 proteins are expressed post-synaptically and form Ca^2+^-dependent, *trans*-synaptic complexes with presynaptic neurexins (Nguyen and Sudhof, 1997; Scheiffele et al., 2000). NLGN2/NRXN interactions are believed to be important for the recruitment and assembly of post-synaptic complexes that support inhibitory signaling through GABA_A_ receptors (Graf et al., 2004; Poulopoulos et al., 2009), though other types of synapses also appear to demonstrate these complexes (Chen et al., 2012; Takacs et al., 2013). Importantly, in addition to NLGN2, we also identified in our SERT proteomic effort several other inhibitory synaptic proteins including GABA_A_ receptor subunits and the GABA_A_ scaffolding protein collybistin. The convergence on NLGN2 of our prior SERT transcriptomic with proteomic effort compelled a further evaluation of the unexpected relationship between transporter and NLGN2. Our findings confirm specific SERT:NLGN2 protein associations that are localized to cell body but not axonal projections of 5-HT neurons. Moreover, molecular, physiological, and behavioral changes evident in *Nlgn2* null mice reveal a reliance on multiple dimensions of 5-HT neuron homeostasis on NLGN2 expression, leading to considerations of altered synaptic adhesion complexes as potentially contributory to disorders linked to perturbed 5-HT signaling.

## Materials and Methods

### Animals and Genotyping

All studies with mice were performed under approved protocols of the Institutional Animal Care and Use Committees of the Vanderbilt University School of Medicine or of the Children’s Hospital of Los Angeles, University of Southern California. Animals were housed on a 12:12 light:dark cycle with lights on at 6:00 AM and food and water provided *ad libitum*. *Nlgn2* null mice (on C57BL/6, 129 mixed background) were obtained from Jackson Labs (stock number: 008139). Our colony was derived from four heterozygous breeding pairs and maintained via het × het breeding paradigm. All subject animals were derived from this breeding scheme, except as noted below. Mouse genotyping was performed on genomic DNA extracted from tail clips using the Extract-N-AMP tissue PCR kit (Sigma, St. Louis, MO, USA) according to the manufacturer’s instructions. Genotyping was performed by PCR with oligonucleotides 5′ GTC TCA GTA AGC TTA TTT GAG AAG CCA A 3′ (RB4485), 5′ CTC TGG GCC TTC TCA GGA CTG TAC 3′ (RB4486), and 5′ GAG CGC GCG CGG CGG AGT TGT TGA C 3′ (RB4487), based on Jackson Labs protocol. Genotypes were determined by the presence or absence of WT (582 bp) and mutant (565 bp) bands, as determined by agarose gel electrophoresis.

### Antibodies

SERT: guinea pig anti-5-HTT (#HTT-GP-Af1400, Frontier Institute, Japan; 1:2,000 for western blots); rabbit anti-SERT (#Pc177L, CalBioChem, Billerica, MA, USA, 1:1,000 for immunohistochemistry); SERT anti-serum #48 (see Qian et al., 1995, for co-immunoprecipitation). NLGN2: mouse anti-NLGN2 (#129211, Synaptic Systems, Germany, 1:1,000 for western blots); rabbit anti-NLGN2 (#129203, Synaptic Systems, Germany, 1:5,000 for immunohistochemistry); goat anti-NLGN2 (#sc-14089, Santa Cruz, Dallas, TX, USA, for co-immunoprecipitation). Neurexin: anti-NRXN (#175003, Synaptic Systems, Germany, 1:1,000 for western blots). Collybistin: anti-CB (#261003, Synaptic Systems, Germany, 1:1,000 for western blots). Dopamine transporter (DAT): mouse anti-DAT (generously provided by Dr. Roxanne Vaughan, University of North Dakota School of Medicine, 1:2,000 for western blots, Vaughan et al., 1993). Norepinephrine transporter (NET): mouse anti-NET (NET05, Mab Technologies, Stone Mountain, GA, USA, 1:1,000 for western blots). β-actin: mouse anti-actin (#A3854; Sigma-Aldrich, St. Louis, MO, USA; 1:10,000 for western blots). HRP or fluorophore conjugated secondary antibodies were purchased from Jackson ImmunoResearch (West Grove, PA, USA).

### Western Blotting and Co-immunoprecipitation

For western blotting, freshly dissected brain tissues were homogenized in RIPA buffer (50 mM Tris, pH 7.4, 150 mM NaCl, 1 mM EDTA, 1% TRITON X-100, 1% sodium deoxycholate, 0.1% SDS) containing protease inhibitors (P8340, 1:100; Sigma, St. Louis, MO, USA). Protein concentrations were determined by the BCA method (ThermoFisher, Waltham, MA, USA). Protein lysates were centrifuged for 15 min at 16,060 × *g* and equal amounts of supernatants were separated by 10% SDS-PAGE, blotted to PVDF (Bio-Rad, Hercules, CA, USA) membrane and then incubated with primary and secondary antibodies at dilutions noted above. Immunoreactive bands were identified by chemiluminescence (Western Lightning ECL Pro, Perkin Elmer) and X-ray film (Biomax MS, Kodak, 8294985) exposure. Multiple exposures were obtained to ensure linearity of data capture. For SERT co-immunoprecipitation assays, protein A Dynabeads (Invitrogen) were first cross-linked with an anti-SERT serum #48 using dimethyl pimelimidate (DMP; Schneider et al., 1982). Briefly, beads were washed 3X in PBS at room temp followed by incubation with #48 anti-SERT serum at 4°C for 1 h. Antibody-bound beads were then incubated with 6.5 mg/mL DMP in 0.2 M Triethylamine (TEA) buffer for 30 min at room temperature. The incubation step was repeated 3X with freshly made DMP buffer each time. Cross-linked beads were then quenched in 50 mM ethanolamine (EA) buffer for 5 min at room temp, washed 2X in 1 M glycine (pH = 3) buffer, and then 3X in PBS (10 min, room temp). For NLGN2 co-immunoprecipitation, protein G Dynabeads (Invitrogen) were washed 3X in PBS before incubation with anti-NLGN2 polyclonal antibody (Santa Cruz) for 1 h at room temperature. Beads were washed 3X in PBS buffer before use. For all co-immunoprecipitation assays, brain tissues were homogenized in homogenization buffer (50 mM Tris, 150 mM KCl, 1 mM DTT, 1 mM EDTA, 0.5% *N*-dodecyl-β-D-maltopyranoside, pH 7.4) with protease inhibitors (1:100, Sigma) followed by 1 h incubation at 4°C. Protein lysates were collected by centrifugation at 16,060 × *g* for 10 min. Protein lysates and beads were mixed and incubated at 4°C for overnight, and washed 4X in washing buffer (PBS, 0.1% Triton X-100). Immunocomplexes were eluted by incubating beads with 2X Laemmli sample buffer at 70°C for 10 min. Eluted samples were subjected for SDS-PAGE, electrotransferred to PVDF membranes and Western blotting as noted above.

### Proteomic Evaluation of SERT Complexes

Proteomic analysis was performed in the Vanderbilt Proteomics Core Facility of the Mass Spectromery Research Center. SERT immunocomplexes eluted from the antibody-conjugated dynabeads were first resolved for 6 cm using a 10% Novex precast gel, followed by in-gel tryptic digestion (55°C, 1 h) to recover peptides. Peptides were analyzed via MudPIT (Multidimensional Protein Identification Technology^1^^,^^2^). Briefly, digested peptides were loaded onto a biphasic pre-column consisting of 4 cm of reversed phase (RP) material followed by 4 cm of strong cation exchange (RP) material. Once loaded, this column was placed in line with a 20 cm RP analytical column packed into a nanospray emitter tip directly coupled to a linear ion trap mass spectrometer (LTQ XL, ThermoScientific, Waltham, MA, USA). A subset of peptides were eluted from the SCX material onto the RP analytical column via a pulse of volatile salt, with eluted material then separated by an RP gradient, and then ionized directly into the mass spectrometer. Data derive from eight salt elution steps over the course of approximately 16 h. Both the intact masses (MS) and fragmentation patters (MS/MS) of the peptides were collected and the peptide MS/MS spectral data searched against the mouse protein database using Sequest^3^. Resulting identifications were collated and filtered using IDPicker^4^ and Scaffold^5^.

### *In situ* Hybridization

Adult mice were anesthetized with isofluorane prior to decapitation and brain removal. All brains were immediately frozen in ice-cold isopentane and stored at **-**80°C. Fresh-frozen brains were cryosectioned in the coronal plane at 25 μm and collected on Superfrost Plus glass slides (Fisher, Pittsburgh, PA, USA). Slides were sored at **-**80°C. Commercially available RNAscope ISH Multiplex kits and probes were purchased directly from Advanced Cell Diagnostics, Hayward, CA, USA. ISH probes were: Mm-Nlgn2 (probe region: 3850–4910 bp of GenBank NM_198862.2; C1), Mm-Tph2-C2 (probe region: 1640–2622 bp of GenBank NM_173391.3; C2), and Mm-Slc6a4-C2 (probe region: 452–1378 bp of GenBank NM_010481.2; C2). Each set of target probes contains a tag that enables the target to be visualized in a specific color channel: C1, excitation 495 nm/emission 520 nm and C2, excitation 555 nm/emission 575 nm. *In situ* hybridization was performed according to the manufacturer’s recommendation. Briefly, slides were first post-fixed with pre-chilled freshly made 4% paraformaldehyde for 15 min at 4°C followed by dehydration with 50% ethanol, 70% ethanol, and 100% ethanol at room temp for 5 min each. Dehydrated slides were stored at **-**20°C until the next day for hybridization. Hybridization was performed at 40°C for 2 h in a HybEZ oven (Advanced Cell Diagnostics, Hayward, CA, USA) after pretreating slides with Protease Pretreat 4 (Advanced Cell Diagnostics, Hayward, CA, USA) at room temp for 30 min. After wash and amplification steps, Prolong Gold with DAPI (Life Technology, Carlsbad, CA, USA) was used to mount the coverslips. A multiplex positive control probe mix consisting of probes against three different house keeping genes, Polr2a (C1), Ppib (C2), and Ubc (C3), and negative control probes against a bacterial gene, dapB (all three channels), were purchased from Advanced Cell Diagnostics and were used as references for the signal intensity and background level in each channel. Images were acquired using a Zeiss Axio Observer inverted confocal microscope fitted with an LSM700 confocal scanner (Cellular Imaging Core at the Saban Research Institute at Children’s Hospital Los Angeles). Figures were prepared digitally in Adobe Photoshop CS5.1 (Adobe Systems, Inc., San Jose, CA, USA).

### Immunohistochemistry

Mice used for immunohistochemistry were anesthetized by intraperitoneal pentobarbital injection (65 mg/kg), and subjected to intracardiac perfusion with cold PBS (pH 7.4) followed by 4% freshly prepared paraformaldehyde. Brains were immediately removed, post-fixed in the same fixative at 4°C overnight and then cryoprotected in 30% sucrose. Brains were then frozen and sectioned (40 μm) using a sliding microtome (Leica SM-2000). Free-floating sections were washed 3X in PBS buffer and blocked in PBS buffer containing 0.2% Triton-X and 3% normal donkey serum for 1 h at room temperature. The sections were incubated with primary antibodies overnight at 4°C followed by fluorophore-conjugated secondary antibodies (2 h, room temperature). Images were obtained in the Vanderbilt Cell Imaging Shared Resource using a Zeiss Axio Imager M2 microscope equipped with X-Cite 120 laser source (Lumen Dynamics). Confocal images were obtained using a Zeiss LSM 510 Meta confocal microscope.

### Brain Tissue Neurochemical Measurements

For assessment of 5-HT, 5-HIAA, glutamate, and GABA levels in WT and *Nlgn2* null mice, tissues were homogenized using an Omni Tissue Homogenizer, in 100–750 μl of 0.1 M trichloroacetic acid (TCA), 10 mM sodium acetate, 0.1 mM EDTA, 5 ng/ml isoproterenol (as internal standard), and 10.5% methanol (pH 3.8). 5-HT and 5-HIAA were determined by HPLC through the Neurochemistry Core of the Vanderbilt Brain Institute, utilizing an Antec Decade II (oxidation: 0.5) electrochemical detector operated at 33°C. Twenty μl samples of the supernatant were injected using a Water 717+ autosampler onto a Phenomenex Nucleosil (5 μm, 100A) C18 HPLC column (150 mm × 4.60 mm). Samples were eluted with a mobile phase consisting of 89.5% 0.1 M TCA, 10 mM sodium acetate, 0.1 mM EDTA and 10.5% methanol (pH 3.8). Solvent was delivered at 0.6 ml/min using a Waters 515 HPLC pump. HPLC control and data acquisition were managed by Millennium 32 software. Amino acids were evaluated using the Waters AccQ-Tag system and detected with a Waters 474 Scanning Fluorescence Detector. Ten μl samples of the supernatant were diluted with 70 μL of borate buffer to which 20 μL aliquots of 6-aminoquinol-*N*-hydroxysuccinimidyl carbamate and 10 μL 250 pmol/μL alpha-aminobutyric acid (as internal standard) were added to form the fluorescent derivatives. After heating the mixture for 10 min at 37°, 10 μL of derivatized samples were injected into the HPLC system, consisting of a Waters 2707 Autosampler, two 510 HPLC pumps, column heater (37°C) and the fluorescence detector. Separation of the amino acids was accomplished by gradient elution over a Waters amino acid column and supplied buffers (A – 19% sodium acetate, 7% phosphoric acid, 2% triethylamine, 72% water; B – 60% acetonitrile). HPLC control and data acquisition was managed by Empower 2 software.

### Synaptosomal [^3^H]-5-HT Transport Activity Measurements

Mouse brain synaptosomes were prepared as previously described (Zhu et al., 2011). Briefly, freshly dissected brain tissues were homogenized in 0.32 M sucrose buffer with 10 mM HEPES and 2 mM EDTA (pH = 7). P2 pellets were obtained by centrifugation, resuspended in KRH assay buffer containing 130 mM NaCl, 1.3 mM KCl, 2.2 mM CaCl_2_, 1.2 mM MgSO_4_, 1.2 mM KH_2_PO_4_, 1.8 g/L D-glucose, 10 mM HEPES, pH 7.4, 100 μM pargyline, and 100 μM ascorbic acid, and protein quantified by BCA method (Pierce). Radiolabeled [^3^H]-5-HT uptake into synaptosomes (40 μg) was performed for 10 min at 37°C and terminated using a Brandel Cell Harvester (Brandel), as previously described. Non-specific uptake was determined using parallel samples incubating with 10 μM paroxetine with levels here subtracted from total accumulation to yield specific uptake activity.

### Electrophysiology

#### Whole Cell Recordings

For 5-HT whole cell recordings, *Nlgn2^-/-^* mice were first crossed to a *Tph2*::ChR2-EYFP line (Zhao et al., 2011). The offspring of these breeding were then bred to *Nlgn2^+/-^* mice to generate WT and *Nlgn2^-/-^* mice with YFP-labeled 5-HT neurons. Acute brain slices (190 μm thickness) were prepared in oxygenated ice-cold sucrose-substituted artificial cerebrospinal fluid (aCSF) or NMDG-aCSF^6^ using a vibratome (VT1000S, Leica Biosystems, Nussloch, Germany). The slices were incubated with oxygenated aCSF (124 mM NaCl, 2.8 mM KCl, 2 mM CaCl_2_, 2 mM MgSO_4_, 1.25 mM NaH_2_PO_4_, 26 mM NaHCO_3_, 10 mM D-glucose, pH 7.4) at room temperature for 1 h before using. YFP-expressing serotonergic neurons were chosen under a fluorescence microscope (Axioskope 2 FS, Carl Zeiss MicroImaging, Thornwood, NY, USA). To measure spontaneous inhibitory post-synaptic currents (sIPSCs) in DR neurons, whole-cell voltage clamp (-70 mV) was performed in aCSF at a perfusion rate of 0.5 mL/min at 32°C. Pulled glass pipettes (5 MΩ) were filled with high chloride solution (150 mM KCl, 5 mM NaCl, 10 mM HEPES, 1 mM EGTA, 2 mM MgCl_2_, 2.5 mM Na_2_ATP, 0.2 mM Na_2_GTP, 10 mM D-glucose, pH 7.4). Series resistance was limited to less than 40 MΩ and compensated 90% with a 60 μs lag. Calculated liquid junction potentials (3.6 mV) were not adjusted in our experiments. sIPSC were recorded for 10 min at a 5 kHz sampling rate in the presence of the AMPA receptor antagonist CNQX (10 μM). GABAergic nature of sIPSC was validated using the GABA_A_ receptor antagonist gabazine (10 μM). After high-pass (1 Hz) and low-pass (2 kHz) filtering, sIPSC events were detected by scaled template fitting-method (Clements and Bekkers, 1997) in Clampfit software (Molecular Devices, Sunnyvale, CA, USA). The template was created with accumulation of clearly visible events (>50 events) and the template time course parameters were fitted to the data at template match threshold 4. A Kolmogorov–Smirnov test in Clampfit was performed to compare cumulative distribution histograms. To quantitative 5-HT_1A_ autoreceptor-mediated serotonergic inhibition, 5HT_1A_ agonist-induced hyperpolarization was measured (Beck et al., 2004; Audero et al., 2013). The intracellular solution was composed of 135 mM K-Gluconate, 10 mM KCl, 5 mM HEPES, 0.5 mM EGTANa_4_, 2 mM MgCl_2_, 2.5 mM Na_2_ATP, 0.2 mM Na_2_GTP, pH 7.4). Under whole-cell current-clamp made with +50 pA current injection, we perfused slices with the 5-HT_1A_ receptor agonist 8-OH-DPAT (1 μM) to inhibit serotonergic firing. For analysis, baselines were normalized to the threshold for action potential firing. Recordings were obtained with an Axopatch 200B amplifier (Molecular Devices) connected to a Digidata 1322A Digitizer (Molecular Devices) under the control of a Windows 7-based 32-bit computer equipped with Clampex 10.2 software (Molecular Devices).

#### Multi-neuron Recordings

Array-based, multi-neuron recordings were performed as previously described (Green et al., 2015). Briefly, mice were euthanized by cervical dislocation, brains were extracted and mounted in cold, oxygenated (95% O_2_–5% CO_2_) dissecting media (114.5 mM NaCl, 3.5 mM KCL, 1 mM NaH_2_PO_4_, 1.3 mM MgSO_4_, 2.5 mM CaCl_2_, 10 mM D-glucose, and 35.7 mM NaCHO_3_), and 280 μm thick coronal slices were taken using a Vibroslicer (Campden Instruments). The dorsal raphe nuclei were isolated by removing the extraneous cortical tissue and placed sample in a slice chamber full of room temperature, oxygenated, extracellular recording media (124 mM NaCl, 3.5 mM KCl, 1 mM NaH_2_PO_4_, 1.3 mM MgSO_4_, 2.5 mM CaCl_2_, 10 mM D-glucose, and 20 mM NaHCO_3_). Mid-DR slices of 280 μm thickness were taken between -4.5 and -4.75 mm back from bregma. We used a 6X10 perforated array with electrodes that have a diameter of 30 and 100 μm spacing between electrodes. Once mounted on the array the slice was allowed to settle for at least 45 min. Oxygenated, extracellular recording media (in mM: 124 NaCl, 3.5 KCl, 1 NaH_2_PO_4_, 1.3 MgSO_4_, 2.5 CaCl_2_, 10 D(+)-glucose, and 20 NaHCO_3_) containing 40 μM tryptophan and 3 μM phenylnephrine was perfused over the slice at a rate of 1.3 ml/min during acclimation period as well as during recording. Putative 5-HT neurons were identified by feedback inhibition evoked by application of 8-OH-DPAT (1 μM) for 5 min, with those cells demonstrating a minimum of 50% spike rate suppression being included in the analysis, as has been done in previously published studies (Thompson et al., 2011; Veenstra-VanderWeele et al., 2012). The threshold for detection was set manually to a level that will include all legitimate spikes with the least amount of unipolar noise spikes included (between 13 and 35 μV). Once the total number of spikes was determined for the period of the recording before the application of 5-HT or 8-OH-DPAT that number was divided by the total time in which those spikes occurred to produce a measure of spikes per second.

### Animal Behavior Testing

#### Tail Suspension Test (TST)

Mice were suspended from the edge of a shelf by their tails, secured by an adhesive tape approximately 1 cm away from the tip of the tail. Each trial was conducted for a period of 6 min. Total immobility time was measured by using the program iCheckClock (Sgmultimedia^7^). Mice were deemed immobile when they hung passively and were completely motionless.

#### Forced Swim Test (FST)

Mice were gently lowered into a transparent Plexiglas cylinder (20-cm diameter) filled halfway with water (25 ± 1°C) for a 6-min session. The water was changed after each mouse tested. Total immobility time was measured as in the TST. Mice were deemed immobile when floating motionless or making only necessary movement to keep afloat.

#### DOI-Induced Head Twitch

Mice were administered 1.0 mg/kg of 1-(2,5-dimethoxy-4-idophenyl)-2-aminopropane (DOI; Sigma, St. Louis, MO, USA) in PBS solution via injection (i.p.) in a volume of 10 mL/kg. Thirty-four minutes post-injection, each mouse was placed in a large glass beaker containing their home cage bedding. Two researchers who were blind to genotype independently counted the number of head twitches during a 15-min test period.

#### Tube Test

Tube test assays for social dominance were performed as previously described (Veenstra-VanderWeele et al., 2012). Briefly, a clear acrylic tube 30-cm-long, 3.5-cm-diameter with small acrylic funnels added to each end to facilitate entry into the tube was used. On two separate days before testing, each mouse was exposed to the tube, with progress through the tube resulting in the mouse being returned to the home cage. For the tube test bouts, one *Nlgn2* null and one WT mouse of the same sex and age, but from different home cages, were placed at opposite ends of the tube and released. A subject was deemed a “winner” when its opponent backed out of the tube.

### Statistical and Graphical Analyses

Data from experiments was analyzed and graphed using Prism 5.0 (GraphPad Software, Inc., La Jolla, CA, USA). For all analyses, a *P* < 0.05 was taken to infer statistical significance. Specific details of statistical tests are given in Figure Legends.

## Results

### SERT and NLGN2 form Midbrain-Specific Protein Complexes

To pursue an unbiased approach to identify novel SERT interacting proteins, we immunoprecipitated SERT protein complexes from midbrain lysates prepared from WT and SERT null mice, using beads covalently coupled to SERT anti-serum #48 (Qian et al., 1995). SERT-associated immunocomplexes were eluted through competition binding of the C-terminal SERT peptide #48 was raised against. MudPIT (Multi-dimensional Protein Identification Technology, Wolters et al., 2001) analysis was used to isolate peptides and afford SERT-associated protein identification. From the 853 proteins identified in MudPIT analyses, 45 proteins were identified in WT but not SERT null samples, with an additional 80 proteins showing higher than two-fold enrichment in WT compare to SERT null samples (Supplementary File 1). In the list of proteins, we identified peptides derived from protein phosphatase 2A catalytic subunit, a protein we previously identified as a SERT partner, supporting the utility of our proteomic approach. Also within this group, we identified NLGN2 based on two different peptides (AA 41–50 and 231–243). This finding drew our attention as we had previously identified *Nlgn2* in our study of genes whose expression significantly correlated with midbrain SERT mRNA levels across BXD recombinant inbred lines (Ye et al., 2014a,b). Adding significance to this finding was our recovery of GABA_A_ receptor α2 subunit (two peptides WT, one peptide null). Additionally, several proteins known to be co-localized with NLGN2 at inhibitory synapses (Poulopoulos et al., 2009; Gauthier et al., 2011), including the presynaptic NLGN2 partner NRXN2 and the GDP/GTP exchange factor collybistin, were identified in separate proteomic assays.

**FIGURE 1:**
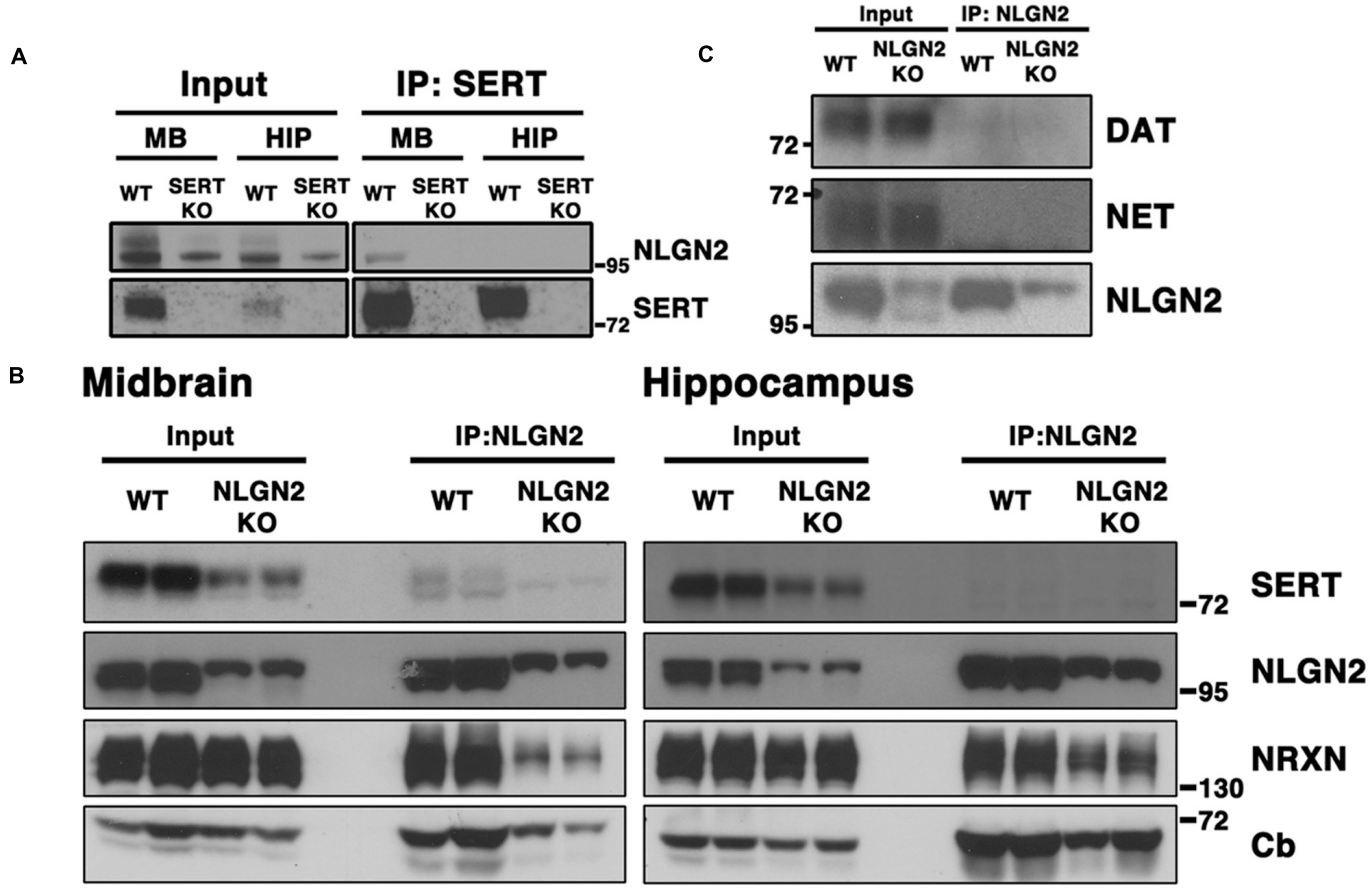
**NLGN2 forms midbrain-specific protein complexes with SERT. (A)** Co-immunoprecipitation assay with SERT antibody, using SERT null mice as negative controls; **(B)** Co-immunoprecipitation assay with NLGN2 antibody, using NLGN2 null mice as negative controls, NRXN: neurexin, Cb: collybistin; **(C)** NLGN2 does not form protein complexes with dopamine transporter (DAT) or norepinephrine transporter (NET). Input lanes represent 1% of total protein lysates, co-IP lanes represent 40% of total eluants. Each blot is a representative of three or more independent experiments.

To validate our proteomic findings, we performed co-immunoprecipitation assays using midbrain extracts from WT and SERT null mice, capturing complexes with SERT or NLGN2 antibodies and blotting for the other protein. As predicted, we obtained clear evidence for the presence of NLGN2 in midbrain SERT complexes using extracts from WT animals, but not with extracts from SERT null mice (**Figure 1A**). Similarly, co-immunoprecipitations of NLGN2 recovered SERT from NLGN2 from WT but not NLGN2 null extracts (**Figure 1B**). We also detected several known NLGN2 interacting proteins in these samples, including α-NRXN and collybistin. Blotting of NLGN2 null mice revealed detection by NLGN2 antibody of proteins other than NLGN2, which prior studies indicates is NLGN3, at a higher molecular weight (Dr. Frédérique Varoqueaux, Max-Planck Institute for Experimental Medicine). Importantly, the NLGN3 species was absent from SERT immunocomplexes, adding further evidence of the specificity of SERT/NLGN2 interactions and suggesting that other NLGN-associated proteins in SERT immunoprecipitates derive from NLGN2. Finally, we investigating the specificity of NLGN2 interactions with respect to other neurotransmitter transporters expressed in the midbrain. Although as expected, DAT and NET proteins were well-represented in total extracts, neither protein was detected in NLGN2 immunoprecipitates (**Figure 1C**).

Although conspicuous due to our prior BXD transcriptome studies, our identification of a midbrain SERT:NLGN2 complex was puzzling because NLGN2 has been well-documented to be a post-synaptic component of inhibitory synapses typically found on somatodendritic membranes (Poulopoulos et al., 2009; Panzanelli et al., 2011), whereas SERT is traditionally considered a marker for axonal membranes of raphe 5-HT neurons. However, SERT is synthesized in midbrain raphe soma and expressed somatodendritically (Hensler et al., 1994; Qian et al., 1995; Lau et al., 2010; Colgan et al., 2012), and thus we reasoned that SERT:NLGN2 associations might derive from an interaction in these compartments. We thus sought to determine whether NLGN2-SERT protein complexes are present in brain regions where SERT is only present on axonal membranes, performing co-immunoprecipitation studies from hippocampal extracts. As shown in **Figures 1A,B**, although both SERT and NLGN2 are readily detected in total hippocampal extracts, SERT:NLGN2 complex were not recovered in these samples, using either SERT or NLGN2 co-immunoprecipitations.

**FIGURE 2:**
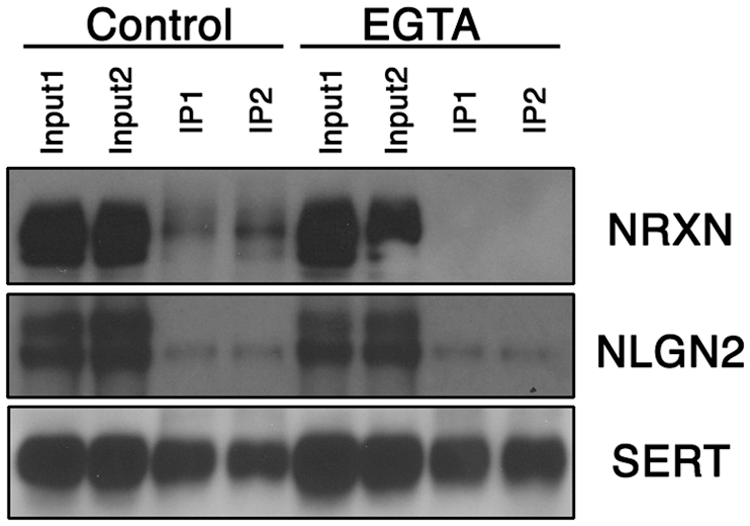
**Midbrain NLGN2:SERT interaction is Ca^2+^-independent.** Co-immunoprecipitation assays using SERT antibody were performed with (EGTA lanes) or without (control lanes) the presence of Ca^2+^ chelator EGTA (10 mM). SERT-bound neurexin was largely removed under Ca^2+^ free conditions, whereas NLGN2 remained bound to SERT, suggesting neurexin is associated with SERT through NLGN2. Input lanes represent 1% of total protein lysates, co-IP lanes represent 40% of total eluents. Blot is a representative of three independent experiments.

The regional specificity of our findings suggested that SERT:NLGN2 complexes might be a feature of the somatodendritic membrane of raphe 5-HT neurons. However, NLGN2 supports interactions between pre- and post-synaptic membranes, and the SERT interactions could arise either in *cis*, where SERT and NLGN2 exist in a complex in the same membrane, or in *trans*, where presynaptic SERT might link to post-synaptic NLGN2 via the transporter’s *cis* interactions with presynaptic NRXN proteins. Indeed, in addition to our identification of NRXN-2 peptides in our proteomic studies, we detected, using a pan-neurexin antibody, α-NRXN, the long splicing isoform of NRXN (Treutlein et al., 2014), in SERT immunoprecipitates (**Figure 2**). Because NLGN:NRXN interactions are Ca^2+^-dependent (Ichtchenko et al., 1996), it is possible to distinguish between *cis* and *trans* interactions of SERT with NLGN2 via treatment of extracts with a Ca^2+^-chelator prior to immunoprecipitation. We found that treatment of midbrain extracts with EGTA treatment prior to SERT immunoprecipitation completely eliminated recovery of α-NRXN without impacting the recovery of SERT or NLGN2. These findings strongly argue for a *cis* model of NLGN2-SERT interactions where NLGN2 and SERT are complexed on somatodendritic membranes across from presynaptic terminals that elaborate α-NRXN, leading to co-immunoprecipitation of α-NRXN with SERT due to transporter:NLGN2 interactions.

### NLGN2 is Expressed in Dorsal Raphe 5-HT Neurons and Co-localizes with SERT on 5-HT Cell Bodies

**FIGURE 3:**
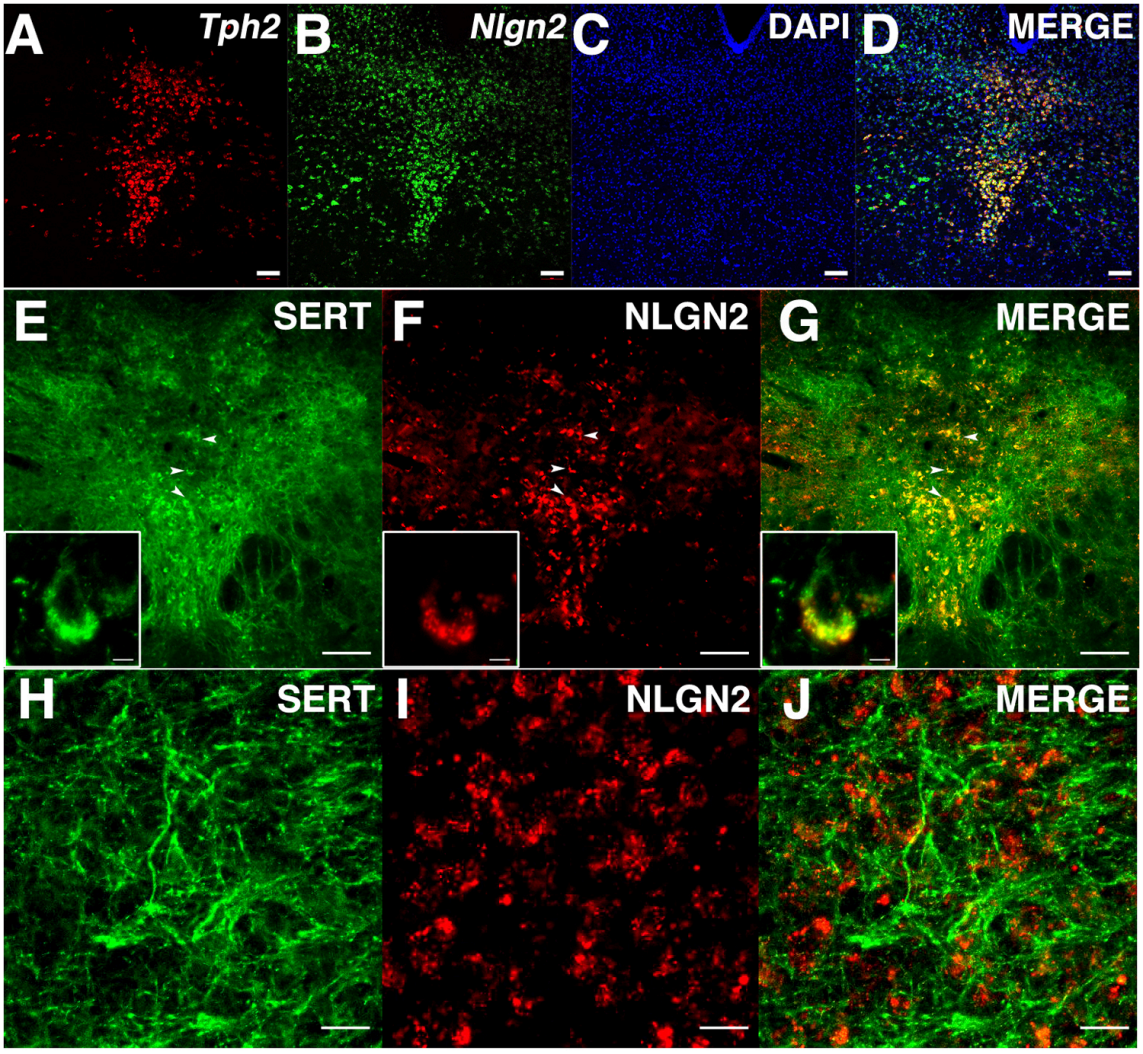
**NLGN2 mRNA and proteins are expressed in the dorsal raphe nucleus (DRN) and co-localize with SERT in 5-HT cell bodies. (A–D)** *Nlgn2* mRNA co-localizes with *Tph2* gene expression in DRN 5-HT neurons (scale bar: 100 μm); **(E–G)** NLGN2 proteins co-localize with SERT on 5-HT cell bodies in DRN (arrowheads, scale bar: 100 μm). Insets: confocal image of NLGN2:SERT co-localization on the cell body a single 5-HT neuron (scale bar: 5 μm); **(H–J)** SERT expressed on midbrain axonal fibers of 5-HT neurons but does not co-localize with NLGN2 (scale bar: 5 μm).

Although our biochemical studies provide strong evidence for expression of NLGN2 by dorsal raphe 5-HT neurons, no previous studies have been published that define sites of expression of NLGN2 in the midbrain. To visualize the expression of the *Nlgn2* mRNA, we used a multicolor *in situ* hybridization approach (RNAscope, ACD), determining the pattern of *Nlgn2* mRNA localization in parallel with that of *Tph2* as a marker of 5-HT neurons. *Tph2* labeling displayed the well-known distribution of 5-HT neurons in the DRN (**Figure 3A**). We found *Nlgn2* mRNA to be more broadly expressed, though not ubiquitously (see DAPI probe, **Figures 3B,C**). Importantly, essentially all *Tph2* positive soma were double labeled with the *Nlgn2* probe (**Figure 3D**). To confirm these findings at the protein level, we performed multi-color immunofluorescence labeling of DRN sections with SERT and NLGN2 antibodies. Consistent with our mRNA studies, we readily detected double labeling of cells with SERT and NLGN2 in DRN cell bodies (**Figures 3E–G**) whereas little or no co-localization was evident in axon profiles coursing through the surrounding neuropil (**Figures 3H–J**). Staining of raphe cells was dramatically diminished in sections from NLGN2 KO mice (data not shown). At higher resolution, somatodendritic co-labeling appeared punctate, either representing sites of surface expression or submembraneous biosynthetic compartments. Although additional studies are needed to explore potential subsets of serotonergic neurons supporting NLGN2 expression, these observations are consistent with the model that dorsal raphe SERT:NLGN2 interactions occur *in cis* on the somatodendritic membrane and not in axonal compartments, either in the midbrain or forebrain projection areas.

### SERT Expression is Reduced in Constitutive NLGN2 Null Mice

**FIGURE 4:**
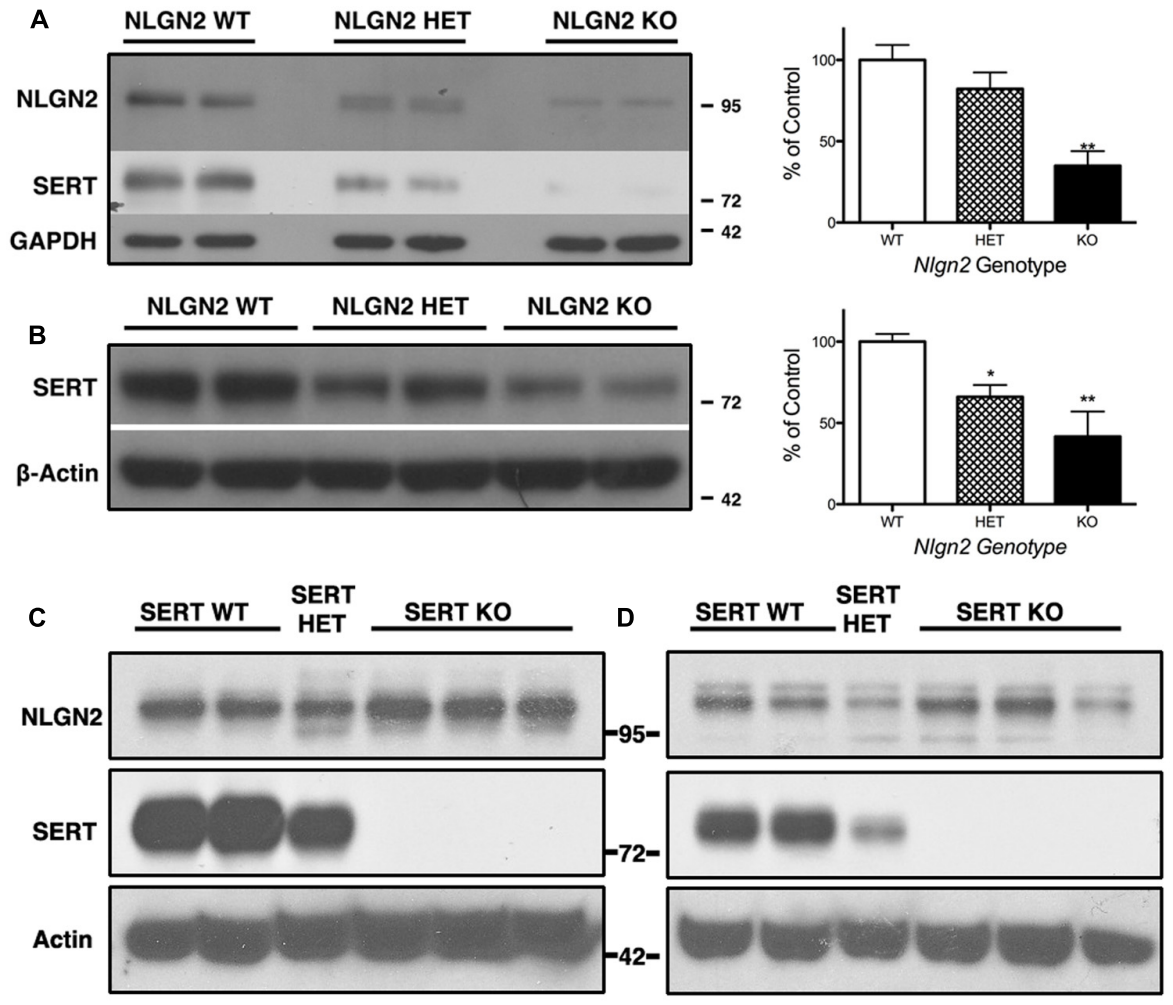
***Nlgn2* null mice exhibit a midbrain-specific reduction in SERT expression.** Western blots of SERT protein in **(A)** midbrain and **(B)** hippocampus. NLGN2 protein levels are not different between SERT null mice and their WT littermates in midbrain **(C)** or hippocampus **(D)**. For **(A)** ^∗∗∗^*P* = 0.001, one-way ANOVA; ^∗∗^*P* < 0.01 for WT vs. null and ^∗^*P* < 0.05 for HET vs. null, Bonferroni’s Multiple Comparison Test, *N* = 5 for each genotype. For **(B)** *P* = 0.003, one-way ANOVA; ^∗^*P* < 0.05 for WT vs. HET and WT vs. null, Bonferroni’s Multiple Comparison Test. *N* = 5 for each genotype.

**FIGURE 5:**
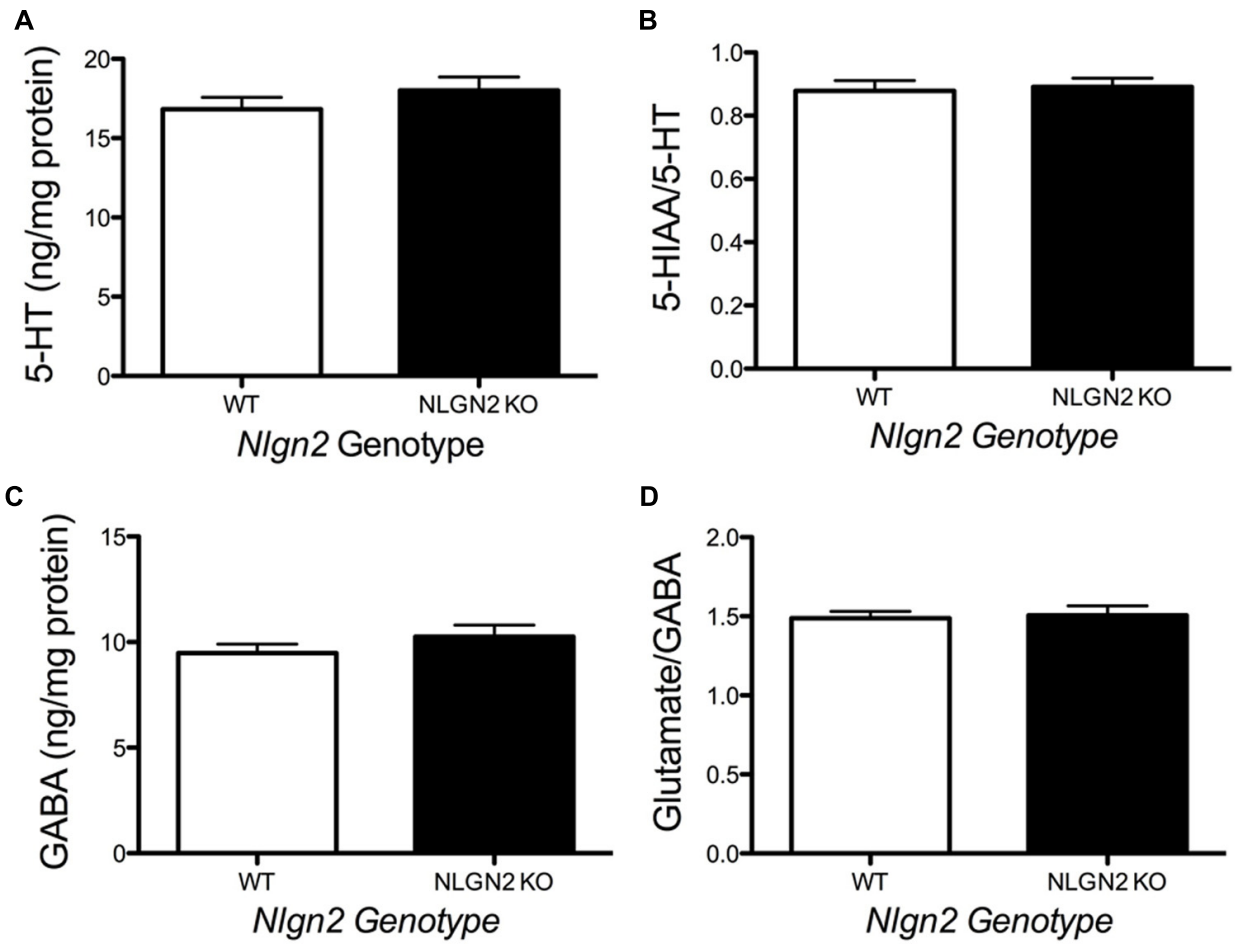
**Loss of NLGN2 expression does not impact steady state 5-HT homeostasis.** Mice have normal midbrain 5-HT levels and 5-HT turnover ratios (**A,B**, *N* = 11 for WT and *N* = 9 for null), as well as GABA levels and glutamate/GABA ratios (**C,D**, *N* = 8 for WT and *N* = 5 for null). *P* > 0.05, Student’s *t*-test.

**FIGURE 6:**
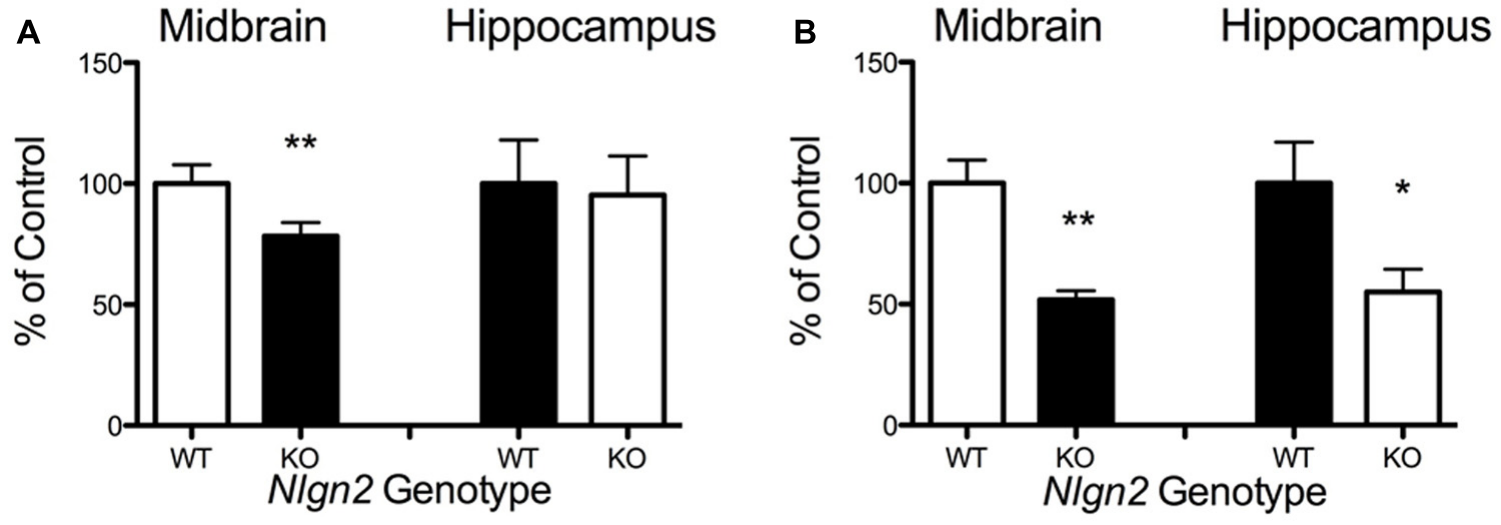
***Nlgn2* null mice exhibit decreased midbrain 5-HT synaptosomal uptake. (A)** Substrate concentration [5-HT] = 50 nM (^∗∗^*P* < 0.01 for midbrain, NS for hippocampus, *N* = 6 for each genotype, Student’s *t*-test); **(B)** Substrate concentration [5-HT] = 1 μM (^∗∗^*P* < 0.01 for midbrain, ^∗^*P* < 0.05 for hippocampus, *N* = 4 for each genotype, Student’s *t*-test).

Our previous BXD studies revealed a strong positive correlation between *Slc6a4* and *Nlgn2* mRNA variation. To test whether this relationship holds at the protein level, we evaluated SERT protein expression in *Nlgn2* null mice. In western blots, we observed a significant *Nlgn2* gene-dosage dependent decrease of SERT protein levels in midbrain extracts (**Figures 4A,B**), with *Nlgn2* null mice displaying a more than 50% of reduction in transporter protein compared to their WT littermates (see also **Figure 1B**), and *Nlgn2*^+/-^ extracts demonstrating intermediate SERT levels. Interestingly, we also found equivalent SERT protein reductions in hippocampal extracts, where we do not recover SERT:NLGN2 complexes. These observations suggest that loss of SERT protein expression arises from an indirect impact of loss of NLGN2 that ultimately influences raphe neuron biosynthetic capacity for SERT and possibly other molecules. To determine whether the protein downregulation is reciprocal between NLGN2 and SERT, we also quantified NLGN2 expression in SERT null mice and their WT littermate controls and found that NLGN2 expression levels did not differ between WT and SERT null mice (**Figures 4C,D**). We could not detect any gross differences in dorsal raphe 5-HT neuron numbers or morphology, as visualized by 5-HT staining (data not shown) and midbrain levels of 5-HT and 5-HT turnover (5-HIAA/5-HT), as assessed by HPLC analysis of tissue extracts, were normal (**Figure 5**). To determine whether loss of SERT protein in *Nlgn2* null mice impacts nerve terminal 5-HT uptake capacity, we measured uptake of radiolabeled 5-HT uptake in midbrain and hippocampal synaptosomes, detecting significant reductions at a saturating substrate concentration (1 μM, **Figure 6**). Altogether, these findings reveal a dependence of SERT expression and activity on NLGN2 levels, likely arising from the changes in SERT mRNA expression predicted by our BXD studies (Ye et al., 2014a,b).

### NLGN2 Null Mice Display Altered 5-HT Neuron Physiology

**FIGURE 7:**
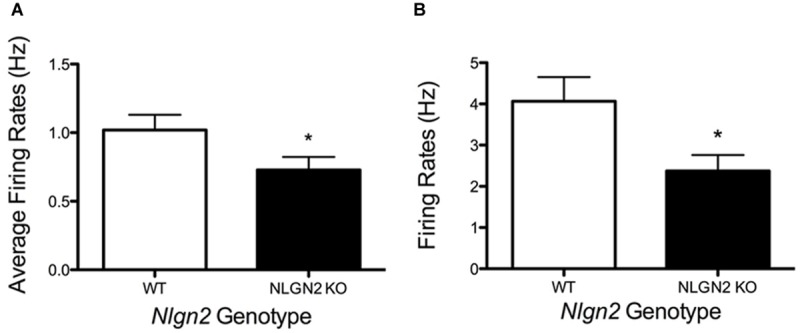
**Changes in dorsal raphe neuron firing rates in *Nlgn2* null mice. (A)** Reduced basal firing rates of 5-HT neurons in dorsal raphe nucleus in multi-electrode array recordings (^∗^*P* < 0.05, *N* = 7 animals and 23 cells for each genotype, Student’s *t*-test). **(B)** Reduced firing rates following +50 pA current injection in whole-cell current clamp of dorsal raphe 5-HT neurons (^∗^*P* < 0.05, *N* = 8 cells for WT and 10 cells for null, Student’s *t*-test).

**FIGURE 8:**
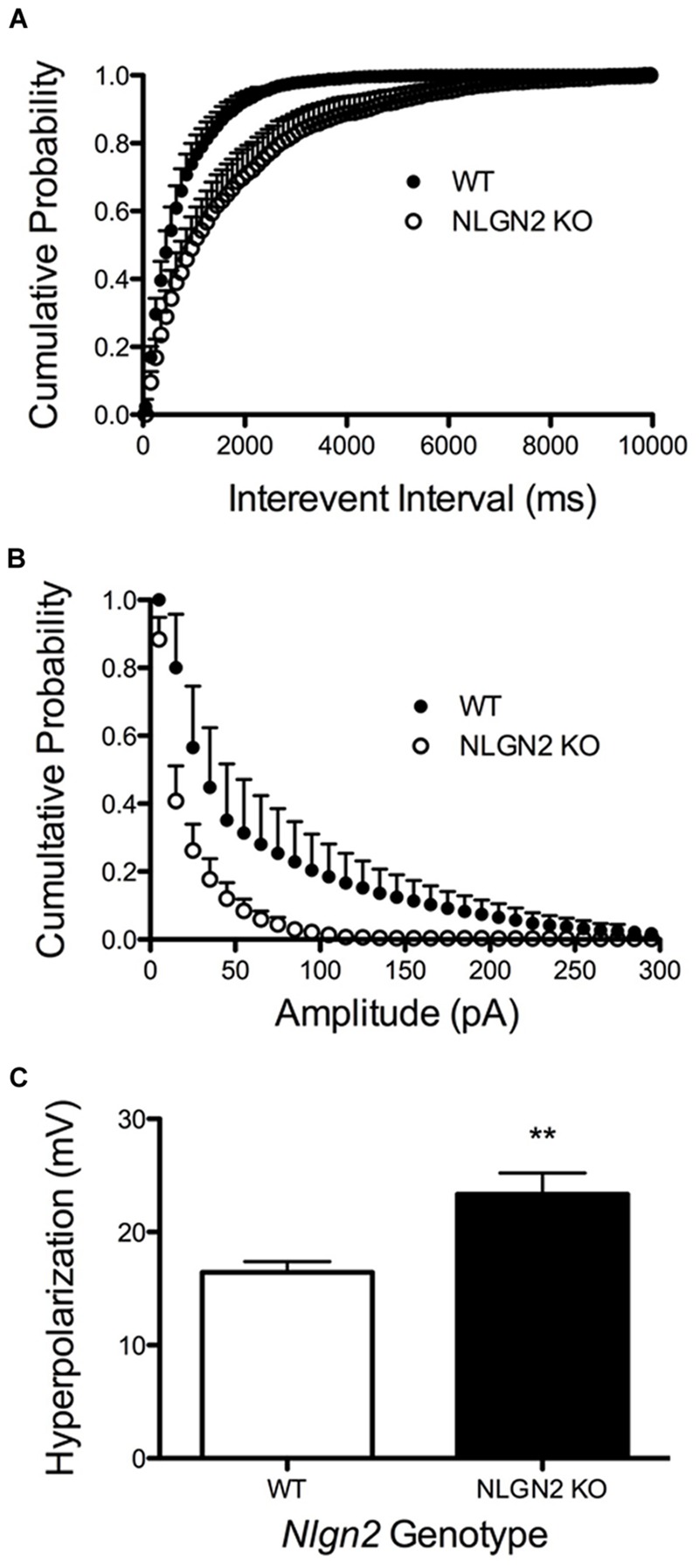
**Altered 5-HT neuron modulation by GABA and 5-HT inputs in *Nlgn2* null mice.** *Nlgn2* null mice exhibit reduced GABA_A_-mediated, gabazine-sensitive sIPSCs, in both **(A)** inter-event interval (*P* < 0.001, *D* = 0.4851, *N* = 5 cells for WT and six cells for null, Kolmogorov–Smirnov test) and **(B)** amplitude (*P* < 0.001, *D* = 0.5392, *N* = 5 cells for WT and six cells for null, Kolmogorov–Smirnov test). **(C)** *Nlgn2* null mice showed enhanced 5-HT_1A_ receptor agonist (8-OH-DPAT)-induced hyperpolarization (^∗∗^*P* < 0.01, *N* = 11 cells for WT and seven cells for null, Student’s *t*-test).

A loss in SERT levels in the *Nlgn2* null in both the midbrain and hippocampus suggests that NLGN2 is required for 5-HT neurons to maintain specific aspects of their functional identity. To gain an initial evaluation of how loss of NLGN2 could impact 5-HT neuron excitability, we utilized a multi-electrode recording array system (Green et al., 2015) to monitor basal 5-HT neuron activation in acute midbrain slices. In this approach, the array detects the firing of neurons in the slice overlying the dorsal raphe nucleus in response to bath application of the α1-adrenergic receptor agonist phenylnephrine, mimicking the excitatory drive of norepinephrine on raphe 5-HT neurons in the intact brain (Judge and Gartside, 2006). Additionally, dorsal raphe 5-HT neurons were identified through their well-described sensitivity to the 5-HT_1A_ receptor agonist 8-OH-DPAT (Nomikos et al., 1996; Evrard et al., 1999). In this effort, we obtained evidence for a significantly reduced basal firing rate of DRN 5-HT neurons in *Nlgn2* null mice compared to the neurons of WT littermates (**Figure 7A**). These effects could relate to an intrinsic reduction in dorsal raphe neuron excitability or to a reduced efficacy of noradrenergic signaling to firing-regulatory K^+^ channels (Karschin et al., 1991). To explore this issue further, we used single-cell patch-clamp recordings to evaluate cell excitability independent of synaptic input and to assess GABAergic and 5-HT_1A_ mediated inhibitory inputs. To further insure correct identification of 5-HT neurons, we bred *Nlgn2* null mice with a reporter strain that expresses YFP under the control of *Tph2* promoter (Zhao et al., 2011). Current injections were used to drive firing of dorsal raphe neurons. As with phenylnephrine-stimulated firing, we found current injections into dorsal raphe 5-HT neurons to exhibit a reduced firing frequency (**Figure 7B**). We also used this recording format to evaluate changes in GABA- and 5-HT-mediated inhibitory responses. We observed significantly reduced GABA_A_ receptor-mediated IPSCs in NLGN2 null slices compared to slices of WT littermates, with reductions evident in both amplitude and frequency (**Figures 8A,B**). In contrast, dorsal raphe 5-HT neurons displayed an increased efficacy of 5-HT_1A_ receptors, captured as an increase in membrane hyperpolarization achieved with a saturating agonist (8-OH-DPAT) concentration (**Figure 8C**).

### *Nlgn2* Null Mice Exhibit Altered Behaviors in Tests Associated with 5-HT Signaling through SERT and 5-HT Receptors

**FIGURE 9:**
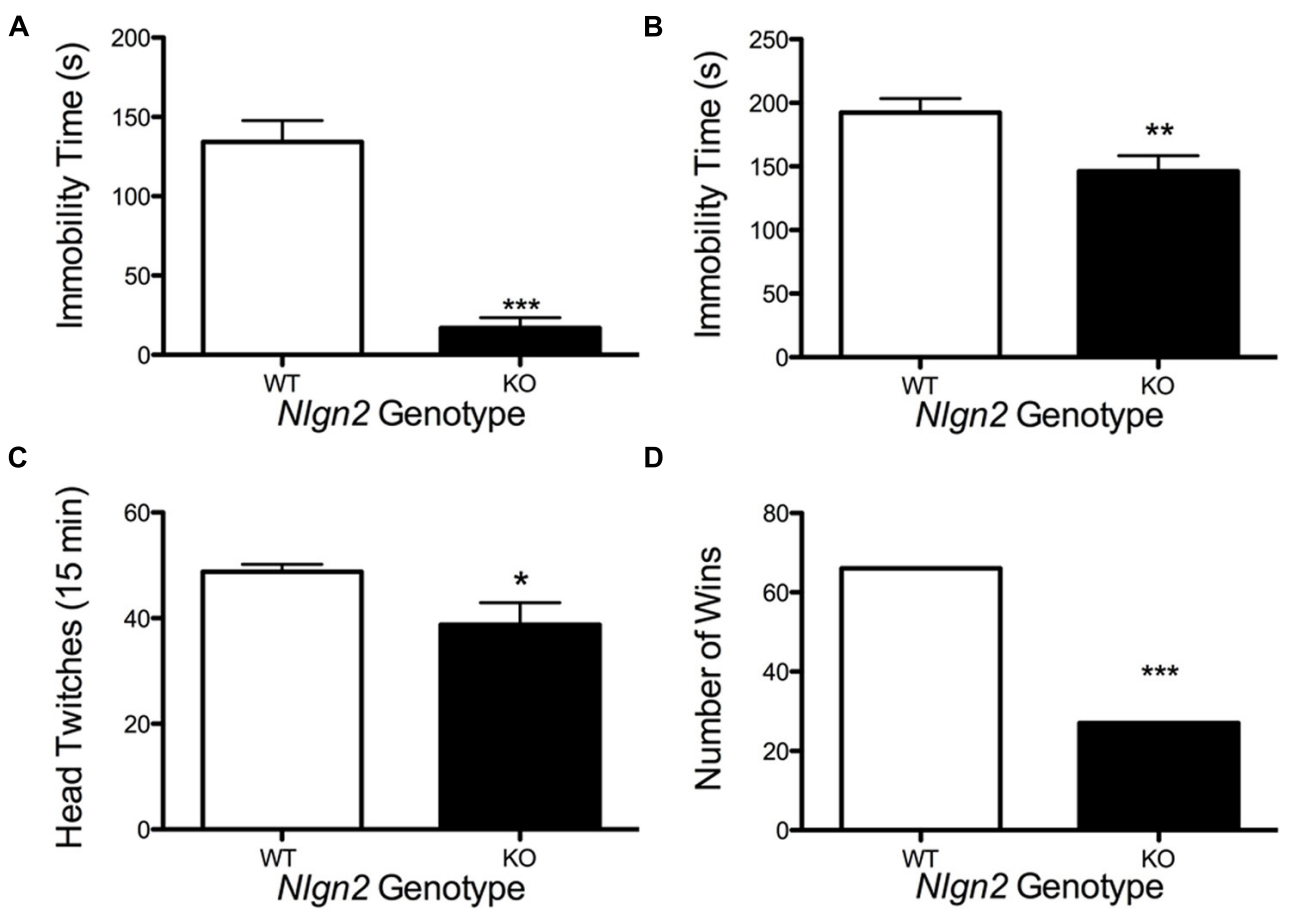
**Altered behavior of *Nlgn2* null mice.** *Nlgn2* null are **(A)** less immobile in the tail suspension (^∗∗∗^*P* < 0.001; *N* = 12 for each genotype, Student’s *t*-test) and **(B)** forced swim test (^∗∗^*P* < 0.01; *N* = 10 for each genotype, Student’s *t*-test), **(C)** have fewer head twitches after 5-HT_2A/2C_ agonist (DOI) injection (^∗^*P* < 0.05; *N* = 6 for each genotype, Student’s *t*-test), and **(D)** back out more in the tube test (^∗∗∗^*P* < 0.001; *N* = 12 for each genotype, McNemar’s test) compared to WT littermates.

*Nlgn2* null mice have been assessed in multiple behavioral tests and found to demonstrate phenotypes suggestive of anxiety (Blundell et al., 2009; Wohr et al., 2013; Babaev et al., 2015) or autism spectrum disorder (ASD; Hines et al., 2008; Kohl et al., 2015; Liang et al., 2015). Owing to the changes observed in the *Nlgn2* null mice in SERT expression and DRN electrophysiological properties, we sought to determine whether these animals would also display changes in behaviors sensitive to pharmacological or genetic SERT manipulation. Reduced immobility in the tail suspension test (TST) and forced swim test (FST) arise in animals with antidepressant-induced blockade of SERT (Holmes et al., 2002). As *Nlgn2* null mice have reduced SERT levels, and we therefore predicted that these mice might display a phenotype similar to that seen with SSRI treatment. Indeed, *Nlgn2* null mice displayed significantly reduced immobility time compared to their WT littermate controls, with the most striking reduction evident in the TST (**Figures 9A,B**). SERT null mice (Rioux et al., 1999; Li et al., 2003) display reduced 5-HT_2A_ receptor expression/sensitivity, presumably arising from elevated tonic extracellular 5-HT exposure. Consistent with these findings, when we queried the sensitivity of post-synaptic 5-HT_2A_ receptors via the DOI-induced head twitching assays, we found that *Nlgn2* null mice had significantly reduced responses compared to WT mice, consistent with diminished or desensitized 5-HT_2A_ receptors (**Figure 9C**). Recently, we have shown that social dominance in the tube test is impacted by expression of an autism-associated SERT coding variant (Veenstra-VanderWeele et al., 2012), and SERT null mice demonstrate reduced social dominance (Lewejohann et al., 2010). Although other tests have demonstrated typical social behaviors in other paradigms, in the *Nlgn2* null mice, we found these animals to also exhibit a significantly higher tendency to withdraw from the tube when encountering a WT mouse (**Figure 9D**).

## Discussion

Our work brings to light the existence of a novel SERT-associated protein complex, one that our biochemical and imaging studies indicates is specifically associated with the somatodendritic compartment of dorsal raphe 5-HT neurons and that contains the synaptic adhesion protein NLGN2. NLGN2 is one member of a family of homologous, synaptic cell adhesion proteins (NLGNs 1–4) that form *trans*-synaptic interactions with presynaptic neurexin (NRXN) proteins. NLGN2 has been consistently found to localized to the post-synaptic side of inhibitory, GABAergic synapses (Graf et al., 2004) as well as a subset of glycinergic synapses (Poulopoulos et al., 2009). A recent electron microscopy immunocytochemical study reported that NLGN2 also exists post-synaptic to cholinergic terminals in the CNS (Takacs et al., 2013). Although we cannot exclude potential interactions of SERT with cholinergic receptor complexes, we obtained evidence in our proteomic and SERT co-immunoprecipitation studies for multiple proteins linked to the function of GABAergic inhibitory signaling, including collybistin and GABA_A_ receptors, and thus we believe our SERT:NLGN2 findings speak to physical proximity and interactions of SERT with determinants of GABAergic modulation of dorsal raphe excitability. Local GABAergic interneurons provide significant inhibitory control of dorsal raphe firing and these neurons receive extensive descending inputs, for example from the medial prefrontal cortex (Celada et al., 2001; Srejic et al., 2015). GABAergic interneurons provide a path through which drugs of abuse including opiates (Tao and Auerbach, 2002) and nicotine (Hernandez-Vazquez et al., 2014) can modulate raphe 5-HT neuron output. Projection neurons, for example from the striatum, also innervate dorsal raphe neurons (Pollak Dorocic et al., 2014; Weissbourd et al., 2014). Several subunits of GABA_A_ receptors, including alpha1–3, were found to be highly expressed by raphe 5-HT neurons (Corteen et al., 2015). The role of NLGN2 as an inhibitory post-synaptic cell adhesion protein that can facilitate organization of GABA_A_ receptor complexes is consistent with our finding of a dramatic loss of 5-HT neuron GABAergic IPSCs in *Nlgn2* null mice.

### Nature of the SERT:NLGN2 Complex

Using reciprocal co-immunoprecipitation experiments with SERT and NLGN2 antibodies in WT and null mice, we demonstrate specificity of protein associations. Although DAT and NET can be readily detected in midbrain extracts, no associations with NLGN2 were obtained, revealing a unique relationship with SERT-dependent signaling. Consistent with this finding, we also observed no significant correlation between *Nlgn2* and DAT mRNA (Ye et al., 2014a,b). SERT is synthesized in the somatic comparted and exported for expression on both somatodendritic and axonal membranes (Tao-Cheng and Zhou, 1999; Descarries and Riad, 2012). We observed SERT and NLGN2 immunoreactivity localized to raphe cell bodies whereas SERT-positive axonal profiles in the surrounding neuropil were NLGN2-negative, suggesting that the SERT:NLGN2 complex is a feature of the dorsal raphe somatodendritic membrane. Consistent with this idea, we could not recover SERT:NLGN2 complexes from hippocampal extracts, despite significant recovery of each protein individually, nor do we recover SERT:NLGN2 complexes efficiently from synaptosomes vs. intact tissue preparations. Although the source of 5-HT input is debated (Bagdy et al., 1998; Colgan et al., 2012; Mlinar et al., 2015), somatodendritic SERT proteins likely work in concert with axonal SERTs to limit feedback inhibition of raphe neurons by 5-HT_1A_ autoreceptors (Blier et al., 1998). A somatodendritic SERT:NLGN2 complex implies interactions of the two proteins in *cis*, vs. a *trans* configuration that would apply if axonal SERT interacted with post-synaptic NLGN2 via the intermediary of a *cis* NRXN interaction. Additionally, we found SERT:NLGN2 complexes to be insensitive to Ca^2+^ chelation, whereas recovery of α-NRXN from SERT immunoprecipitations was lost. Thus, recovery of NRXN proteins with SERT immunoprecipitations is likely an indirect consequence of Ca^2+^ independent associations SERT makes with NLGN2. Recovery of α-NRXN in SERT co-immunoprecipitations also indicates that the SERT:NLGN2 complex is unlikely to represent a transient, intracellular complex that disperses upon sorting of transporter to plasma membrane domains, though higher resolution localization methods are needed to fully resolve this issue and can provide information as to whether SERT forms a complex with NLGN2 early during synthesis and export to the plasma membrane. Although we do not know whether the SERT:NLGN2 interaction is direct, our preliminary experiments indicate that the two proteins can be co-immunoprecipitated from co-transfected HEK-293 cells, suggesting that no neuron-specific scaffolding proteins are required to support the interaction. Further studies with the latter system, as well as candidate interacting domains *in vitro*, are needed to define key structural elements that support SERT:NLGN2 interactions and allow for a further dissection of their functional significance.

### SERT Expression Levels are NLGN2-Dependent

Changes in the levels of major synaptic proteins in the *Nlgn2* null mice have previously been profiled (Blundell et al., 2009). At the whole brain level, elimination of *Nlgn2* expression does not cause significant changes in the expression of proteins that are essential for glutamate and GABA actions at pre- or post-synaptic sites. Thus we were surprised to find a strong positive correlation for mRNA levels of *Nlgn2* and *Slc6a4* in the midbrain of BXD mice. Consistent with these findings, we found that SERT proteins are significantly down-regulated in the *Nlgn2* null midbrain, with intermediate effects observed in *Nlgn2*^+/-^ animals. As these effects did not arise in the context of a commensurate loss of 5-HT neurons or 5-HT levels, they do not derive from an impact of NLGN2 on raphe neuron viability. We also observed no loss of NLGN2 levels in *Slc6a4* null mice, although the fraction of NLGN2 in 5-HT neurons is likely but a small fraction of midbrain expression. Thus, although NLGN2 may impact the stability and export of SERT proteins, it is possible that the correlation of *Slc6a4* and *Nlgn2* mRNA and the loss of SERT protein in *Nlgn2* null mice derive from a disruption of GABAergic (and possibly cholinergic) synaptic input to 5-HT neurons. In this regard, Lukkes et al. (2013) observed decreased *Tph2* mRNA expression with treatment of mice with anxiogenic GABA_A_ inverse agonist treatment, though this short-term treatment (4 h) was insufficient to impact SERT mRNA levels. We also demonstrated a change in intrinsic excitability of serotonergic dorsal raphe neurons in *Nlgn2* null mice indicating changes in the machinery supporting expression of other ion channels whose identity we have yet to pursue. Finally, it is possible that the relationship between *Nlgn2* and *Slc6a4* may be non-cell autonomous, derived from GABAergic modulation of other projections that impinge on 5-HT neurons. In this regard, conditional elimination of *Nlgn2* has been achieved (Liang et al., 2015), providing a path to determining whether the levels of NLGN2 in 5-HT neurons are directly connected to these findings.

### DRN 5-HT Neuron Excitability is Altered in NLGN2 Null Mice

The firing of DRN 5-HT neurons is under control of extrinsic glutamatergic, GABAergic, noradrenergic, and peptidergic inputs (Soiza-Reilly et al., 2013; Weissbourd et al., 2014), as well as intrinsic 5-HT auto-regulation (Blier et al., 1998). As expected, a direct consequence of loss of NLGN2 in 5-HT neurons was a significant reduction in GABAergic IPSCs. The reduction we observe in IPSC amplitude indicates either a desensitization or down-regulation of GABA_A_ receptors, consistent with the function of NLGN2 in organizing GABAergic post-synaptic protein complexes (Graf et al., 2004). Similar effects were also observed in pyramidal neurons from the barrel cortex of NLGN2 null mice (Gibson et al., 2009). Interestingly, the frequency of the spontaneous IPSCs is also reduced in the NLGN2 null mice, suggesting a decrease in GABA release from presynaptic vesicles. This effect may arise from compensatory changes in presynaptic GABAergic terminals communicated through unliganded NRXN proteins. Alternatively, the change in frequency may result from a dispersal of post-synaptic GABA_A_ receptors, reducing the probability that vesicles will be opposed to sufficient receptors to mediate an IPSC response. We also observed changes in DRN 5-HT_1A_ receptor sensitivity in the *Nlgn2* nulls. Reductions in SERT levels that arise in the *Nlgn2* null mice could allow for increased stimulation of 5-HT_1A_ receptors by extracellular 5-HT and increased feedback inhibition. *Slc6a4* null mice display a reduction in the expression of dorsal raphe 5HT_1A_ receptors (Fabre et al., 2000; Li et al., 2000), though more recent work indicates that compensatory mechanisms are engaged to sustain autoreceptor-mediated inhibition (Araragi et al., 2013). How the SERT:NLGN2 complex supports or limits the transporter’s ability to limit 5-HT_1A_ activation will require the development of tools (e.g., interfering peptides) that can disrupt this complex without inducing other changes attendant to a loss of NLGN2 levels. Regardless, NLGN2 appears to be physically poised to play a significant role in coordinating the balance of intrinsic and extrinsic inhibitory control of dorsal raphe 5-HT neurons.

### Potential Translational Relevance of Serotonergic Expression of NLGN2

Rare variation in *NLGN2* has been identified in subjects with schizophrenia (Sun et al., 2011). A 17p13.1 duplication that harbors the *NLGN2* gene has been observed in individuals with intellectual disability (Belligni et al., 2012; Kuroda et al., 2014; Mooneyham et al., 2014). NLGN2 null and overexpressing mice exhibit multiple traits thought to mimic ASD, including communication deficits, as revealed by reduced ultrasonic vocalizations, repetitive behaviors and impaired social interactions (Hines et al., 2008; Blundell et al., 2009; Wohr et al., 2013). Although NLGN2 is expressed throughout the CNS, changes in 5-HT and SERT have been implicated in these disorders as well (Hranilovic et al., 2000; Ozaki et al., 2003; Prasad et al., 2009; Blasi et al., 2013), and our current studies raise the possibility that components of behavioral deficits observed in preclinical models and humans with NLGN2 mutations may derive from compromised 5-HT signaling. The presence of SERT in NLGN2 complexes unique to raphe neurons vs. axonal projections raises the possibility of targeting this subset of transporters pharmacologically with potentially unique clinical benefit.

## Conclusion

Disrupted 5-HT signaling has been associated with many neuropsychiatric disorders including depression, OCD and autism. SERT proteins govern the rate and extent of 5-HT clearance and are organized in the plasma membrane as multi-protein complexes. We have identified a unique interaction between SERT and the *trans*-synaptic adhesion protein NLGN2, with evidence that this interaction arises specifically in raphe 5-HT somatodendritic elements and involves other components of GABAergic inhibitory signaling. Along with evidence that NLGN2 protein levels dictate SERT expression levels, the presence of the SERT:NLGN2 complex reinforces a close relationship between GABAergic signaling mechanisms and biochemical, cellular, and behavioral phenotypes associated with 5-HT neurons.

## Author Contributions

RY and RB conceived project. RY, MQ, HI, H-HW, NG, CJ, DM, JV-V, PL, and RB designed experiments. RY, MQ, HI, H-HW, NG, and CJ performed experiments. RY, MQ, HI, H-HW, NG, CJ, DM, JV-V, PL, and RB analyzed data. RY, MQ, and RB wrote the paper. All authors commented and contributed to the manuscript.

## Conflict of Interest Statement

The authors declare that the research was conducted in the absence of any commercial or financial relationships that could be construed as a potential conflict of interest.

## Footnotes

1 http://www.pnas.org/content/99/12/7900.short

2 http://www.jlr.org/content/53/3/379.short

3 http://pubs.acs.org/doi/abs/10.1021/ac00104a020

4 http://www.ncbi.nlm.nih.gov/pubmed/19522537

5 http://www.proteomesoftware.com

6 www.brainslicemethods.com

7 https://software.com/mac/productivity/icheckclock/2.0.2
